# Antiadipogenesis of *Oroxylum indicum* (L.) Kurz Extract via PPAR*γ*2 in 3T3-L1 Adipocytes

**DOI:** 10.1155/2020/6720205

**Published:** 2020-01-05

**Authors:** Tanaporn Hengpratom, Apichart Ngernsoungnern, Piyada Ngernsoungnern, Gordon Matthew Lowe, Griangsak Eumkeb

**Affiliations:** ^1^School of Preclinic, Institute of Science, Suranaree University of Technology, Nakhon Ratchasima 30000, Thailand; ^2^School of Pharmacy and Biomolecular Sciences, Liverpool John Moores University, Liverpool L3 3AF, UK

## Abstract

*Oroxylum indicum* is regarded as a traditional food with medicinal properties and is used widely throughout Asia. It has previously been demonstrated that *O. indicum* extract (OIE) was able to suppress the differentiation of 3T3-L1 preadipocytes to adipocytes. However, the mechanism underlying the antiadipogenesis of this plant has not been fully investigated. The present study aimed to explore the impact of OIE at 50 to 200 *μ*g mL^−1^ on the molecular mechanism involved in the antiadipogenic activity in 3T3-L1 cells at day 0 of their differentiation to adipocytes. The morphology and biochemistry of the cells on day 12 were investigated and compared to the relevant controls. Adiponectin was measured using enzyme-linked immunosorbent assay (ELISA). The mRNA expression of peroxisome proliferator-activated receptor-gamma 2 (PPAR*γ*2), sterol regulatory element-binding proteins 1c (SREBP-1c), fatty acid synthetase (FAS), glucose transporter (GLUT4), and leptin in adipocytes was determined by real-time PCR. The results demonstrated that the OIE at 200 *μ*g mL^−1^ exhibited strongest suppression on intracellular lipid accumulation. The levels of adiponectin were dramatically increased in the untreated adipocytes, whereas significantly decreased in the 200 *μ*g mL^−1^ OIE-treated adipocytes (*P* < 0.05). Expression of the mRNAs revealed that OIE-treated adipocytes at 200 *μ*g mL^−1^ significantly inhibited the expression of PPAR*γ*2 and SREBP-1c and lowered the level of expression of GLUT4, FAS, and leptin compared to the control (*P* < 0.05). These findings suggest that OIE inhibits adipocyte differentiation along with the downregulation of PPAR*γ*2, SREBP-1c, and GLUT4, leading to the decrease in the expression of FAS and adipokine (leptin and adiponectin). Thus, OIE might be developed for hyperlipidemia and obesity prevention.

## 1. Introduction

Obesity is a major health problem and is associated with increasing risk of cardiovascular disease, certain cancers, and type 2 diabetes [1]. The prevalence of obesity is a worldwide problem, with 13% of the adult population reported as being clinically obese [2]. Adipogenesis can be described as a highly regulated process in which stem cells are converted to mature adipocytes through two processes, commitment and differentiation [3]. An increase in the size and number of adipocytes is thought to be important risk factors for the future development of obesity [4, 5]. Moreover, during the development of adipocytes and de novo lipogenesis, various adipokines and transcriptional factors appear to be important in this circumstance [6]. The current strategies for the prevention and treatment of obesity focus on increasing physical activity, reducing calorific intake, and pharmacological treatments such as orlistat [7]. Another approach would be to limit adipogenesis by inhibiting both the proliferation and differentiation of adipocytes. Plant extracts have been screened to identify compounds that can suppress adipogenesis [8]. One such plant extract that has attracted much interest is *Oroxylum indicum.*

*Oroxylum indicum* is a plant that is found throughout Asian countries. It has been used as a traditional medicine for many years [9]. Several studies have demonstrated that crude extract or pure isolated compounds from different parts of *O. indicum* exhibited various pharmacological activities including anti-inflammatory [10] antidiabetic [11], antioxidant [12], and antiadipogenesis [13] activities. The fruit of *O. indicum* contains a mixture of flavonoids, including baicalein and chrysin [14]. Dunkhunthod et al. found that baicalein at 12.5 *μ*M suppressed lipid accumulation and inhibited the pancreatic lipase activity with the IC_50_ values of 159.71 ± 7.15 *μ*Μ in adipocytes [15]. Our previous study revealed that fruits of *O. indicum* extracts (OIE) at 200 *μ*g mL^−1^ exhibited antiadipogenesis property and reduced intracellular lipid levels in the adipocyte. Furthermore, OIE demonstrated a nontoxic effect on cell viability up to the dose of 200 *μ*g mL^−1^ [13]. Also, an animal study suggested that an oral intake of 200 mg kg^−1^ body weight of the OIE for 28 days was safe in diabetic and normal rats [16, 17]. Adiponectin is a hormone secreted from adipocytes. Previous studies have found that the importance of adiponectin is involved in the proliferation and differentiation of 3T3-L1 preadipocytes [18] and human preadipocytes [19]. As a result, it raises expression of C/EBP*α* and PPAR*γ* genes during adipogenesis and consequently increases of lipid accumulation and glucose transportation in adipocytes [18]. Thus, controlling of adiponectin could be a potential target to inhibit adipogenesis.

According to our previous finding, the OIE exhibited the antiadipogenesis effect on 3T3-L1 adipocyte and caused a change of some biochemical components of the cells measured by FTIR. However, there is no report on whether the OIE effects on adipokines are involved in transcriptional regulation. Thus, the aim of the present study was to investigate the potential function of the OIE on adiponectin secretion and explore the molecular mechanism underlying antiadipogenesis effects of the OIE in 3T3-L1 cells.

## 2. Materials and Methods

### 2.1. Plant Extraction and LC-MS/MS Analysis

Fruit of *O. indicum* was collected from the Wang Nam Khiao district, Nakhon Ratchasima province, Thailand. The plant samples were identified by a botanist, Dr. Santi Watthana (School of Biology, Institute of Science, Suranaree University, Thailand). The voucher specimens were kept at the flora of Suranaree University of Technology Herbarium (SOI0808U). The plant extractions were conducted according to a previous study [13].

The phytochemical compounds of *O. indicum* extract were analyzed following the method of Vlaisavljević et al. with some modifications [20]. Briefly, the determination was performed by the Agilent Technologies 1290 series HPLC with Agilent Technologies 6490 series electrospray ionization triple-quadrupole MS/MS and electrospray ionization (ESI). The injection volumes of all samples were 5 *μ*L. The separation was achieved using a Zorbax rapid-resolution high-definition (RRHD) SB-C18 (2.1 mm id × 150 mm, 1.8 *μ*m) (Agilent Technologies) with a thermostat at 25°C, at a flow rate of 0.2 mL min^−1^ of mobile phase whose composition is *A* = 1% aqueous formic acid and *B* = 1% formic acid in acetonitrile. The solvent gradients were started with 30% solvent *B* at 10 min and 100% solvent *B* at 30 min. Quercetin, apigenin, kaempferol, baicalein, and biochanin *A* (0.01 mg mL^−1^) were dissolved with 100% methanol solution and used as standard reference compounds. The OIE (20 mg mL^−1^) was dissolved in 100% of methanol and kept in darkness at 4°C before analysis.

### 2.2. Cell Culture and Treatment

Cell culture was carried out as previously described by Dunkhunthod et al. [15]. Shortly, 3T3-L1 preadipocytes were cultured in Dulbecco's Modified Eagle's medium (DMEM) containing 10% calf bovine serum (CBS) (GIBCO, Grand Island, NY, USA). At two days after confluence (day 0), the cells were stimulated to differentiate with DMEM containing 10% fetal bovine serum (FBS) (Hyclone, Logan, UT, USA), 1.0 *μ*M of dexamethasone (G Bioscience, St. Louis, MO, USA), 0.5 mM of isobutylmethylxanthine, and 1.0 *μ*g mL^−1^ of insulin for two days. From day 4 onwards, the differentiation media were replaced by 10% FBS/DMEM media containing 1.0 *μ*g mL^−1^ of insulin. These media were changed every two days until the cells were harvested. All media contained 100 *μ*g mL^−1^ of streptomycin and 100 U mL^−1^ of penicillin (GIBCO). Cells were maintained at 37°C in a 95% humidified with 5% of CO_2_ atmosphere.

For the treatment of preadipocytes, the cells were seeded in a 6-well plate at the density of 1.5 × 10^5^ cells/well. The cells were allowed to adhere to the plate for 48 h and were then divided into 8 groups: (1) nondifferentiated cells (ND); (2) untreated differentiated cells (control, CON); (3) differentiated cells treated with 0.1% DMSO (vehicle control, V-CON); (4) differentiated cells treated with 1.67 *μ*g mL^−1^ simvastatin (positive control, SIM); (5–8) differentiated cells treated with 50, 100, 150, and 200 *μ*g mL^−1^ OIE, respectively. On day 10 after the cells were differentiated (gauzed by visible lipid droplet >90% of the control group). The lipid droplets and nuclei of nondifferentiated cells and differentiated cells were stained with Oil Red O and Hematoxylin solutions, respectively, as previously described by Hengpratom et al. [13]. The media and the cells were collected for RNA extraction, protein quantification, and immunocytochemistry detection. Each experiment was repeated three times.

### 2.3. Protein Extraction and Immunoblot Analysis

The expression of adiponectin protein in control and OIE-treated cells was determined by western immunoblotting. The cells were washed 3 times with cold 0.1 M phosphate buffer saline (PBS) and were lysed in cold lysis buffer (10 mM Tris-HCl, 150 mM NaCl, 0.5% Triton X-100, 1 mM EDTA, and 100 mM PMSF, pH 7.2) containing protease inhibitor cocktail (Roche, Mannheim, Germany). The protein concentrations were measured using a bovine serum albumin (BSA) protein assay kit following the company description (Thermo Scientific, Rockford, Winnebago, USA). The lysates (20 *μ*g, each) were subjected to SDS-PAGE on 15% polyacrylamide gel. After electrophoresis, the proteins were transferred to nitrocellulose membrane (GE Healthcare, Piscataway, NJ, USA). The membranes were then blocked with 5% skimmed milk in 0.1 M PBS contains 0.1% Tween-20 (PBST) for 1 h at room temperature, followed by washing with PBST. The membranes were subsequently incubated with mouse anti-mouse adiponectin antibody (Abcam, Cambridge, MA, USA) (1 : 1000 in PBS), overnight at 4°C. Negative controls were obtained by omitting the primary antibody or using the preabsorbed one. After extensive washing, the membranes were incubated with HRP-conjugated goat anti-mouse IgG (Santa Cruz, CA, USA) (1 : 2000 in PBS) for 1 h at room temperature, followed by washing with PBST. The antigen-antibody complex was visualized by adding a DAB kit (Vector Laboratories, Burlingame, CA, USA). Analysis of intensities of the adiponectin protein bands was quantified using ImageJ software under the same pixel area. The data were normalized using *β*-actin as an internal control. The experiments were repeated using cells from 3 different individuals.

### 2.4. Determination of Adiponectin Secreted from 3T3-L1 Cells by ELISA

The level of adiponectin secreted from 3T3-L1 adipocytes was performed as previously described by Ngernsoungnern and Ngernsoungnern with some modifications [21]. On day 12, the culture media were collected from control and OIE-treated groups and centrifuged at 10,000 ×g for 10 min at 4°C. Supernatants containing antigens were collected. Subsequently, 100 *μ*L of 100 *μ*g mL^−1^ of the antigen and human adiponectin peptide (Abcam), which served as a standard, was diluted with ELISA coating buffer, coated onto the ELISA plates, and incubated overnight at 4°C. The plates were blocked for nonspecific binding with 0.25% BSA in 0.01 M PBS, with pH 7.2 for 1 h at 37°C, and subsequently incubated with mouse anti-mouse adiponectin antibody (1 : 3000 in PBS). Negative controls were performed by omitting the primary antibody or using the preabsorbed one. The plates were washed twice with PBST and then incubated with HRP-conjugated goat anti-mouse IgG (1 : 3000 in PBS) for 1 h at 37°C, followed by washing. Finally, the tetramethylbenzidine substrate (Sigma-Aldrich, St. Louis, MO, USA) was added for developing a color reaction. The reactions were stopped by adding 1 N H_2_SO_4_, and the color was then read spectrophotometrically at 450 nm. The sensitivity of the assay was 0.1 *η*g mL^−1^. The intra-assay coefficient of variation was 2.4%, and the interassay coefficient of variation was 5.9%.

### 2.5. Immunocytochemistry for Adiponectin

Adiponectin staining was carried out following the method of Ngernsoungnern and Ngernsoungnern with slight modifications [21]. At day 12, 3T3-L1 preadipocytes and adipocytes were collected, washed with cold PBS, fixed for 15 min in cold acetone, and washed with PBST. After blocking with 4% of BSA for 1 h at room temperature, the cells were washed and incubated with an anti-adiponectin antibody (1 : 200 in PBS), overnight at 4°C. After extensive washing, Alexa 488-conjugated goat anti-mouse IgG (1 : 500) was applied to the samples and incubated for 1 h at room temperature. After washing thoroughly with PBS, the cells were double-stained with Hoechst 33342 (nucleus stain) (1 : 3000) for 10 min and washed twice. Finally, the cells were visualized using fluorescence microscopy.

### 2.6. RNA Extraction and mRNA Expression Analysis

The RNA extraction and purification were performed by the RNA, cell miniprep system following the manufacturer protocol (Promega, Madison, WI, USA). The culture medium was removed from cells, and the cells were washed twice with 0.1 M PBS. After that, 250 *μ*L of lysis buffer was added to each well. The cells were transferred to sterile microcentrifuge tubes, added with 85 *μ*L of 100% isopropanol, and then mixed for 5 sec. The cells were subsequently transferred to a ReliaPrep™ Minicolumn and centrifuged at 12,000 rpm for 2 min. The liquid in the collection tubes was discarded, followed by addition of a 500 *μ*L RNA wash solution. The tubes were then centrifuged at 12,000 rpm for 2 min at 25°C. Freshly prepared DNase I (30 *μ*L) was then applied and incubated for 15 min at room temperature. Column wash solution (200 *μ*L) was then added to the ReliaPrep™ Minicolumn and centrifuged at 12,000 rpm for 2 min, followed by adding of 500 *μ*L RNA wash solution and centrifuged at 12,000 rpm for 2 min. The columns were then placed into new tubes, and RNA wash solution (300 *μ*L) was added and then centrifuged at 12,000 rpm for 2 min. The columns were placed into the collection tubes, followed by addition of 15 *μ*L nuclease-free water and centrifugation at 12,000 rpm for 2 min. The purified total RNA was calculated from the concentration obtained at 260 nm using a NanoDrop.

The expression of adiponectin gene levels was conducted by quantitative real-time PCR (RT-PCR). RNA-directed SYBR Green real-time PCR master mix was used in the PCR reaction according to the manufacturer's instructions (Toyobo, Osaka, Japan). Total RNA at 50 *η*g was prepared for the PCR reaction and then subjected to 45 cycles of amplification (denaturation at 95°C for 5 sec, annealing at 60°C for 10 sec, and extension at 74°C for 15 sec). Primers used for amplifying PPAR*γ*2 were 5′-TgT CTC ATA ATg CCA TCA ggT TTg-3′ (forward) and 5′-gAT AAC gAA Tgg TgA TTT gTC TgT-3′ (reverse), for amplifying SREBP-1c were 5′-CTg TTg gTg CTC gTC TCC T-3′ (forward) and 5′-TTg CgA TgC CTC Cag AAg TA-3′ (reverse), and for amplifying FAS were 5′-ATC CTg gCT gAC gAA gAC TC-3′ (forward) and 5′-TgC TgC TgA ggT Tgg AgA g-3′ (reverse). In addition, primers used for amplification of GLUT4 were 5′-ggg TCC TTA CgT CTT CCT TCT-3′ (forward) and 5′-CCT CTg gTT TCA ggC ACT TT-3′ (reverse), of leptin were 5′-ggA TCA ggT TTT gTg gTg CT-3′ (forward) and 5′-TTg Tgg CCC ATA AAg TCC TC-3′ (reverse), and of *β*-actin, which was used as the reference gene, were 5′-ACA TCT gCT ggA Agg Tgg AC-3′(forward) and 5′-ggT ACC ACC ATg TAC CCA gg-3′ (reverse). All gene expression results were expressed as fold changes of threshold cycle (Ct) value relative to controls using the 2^−ΔΔCt^ methods.

### 2.7. Statistical Analysis

All the data were expressed as the mean ± standard deviation of the mean (mean ± SD). The difference values between adiponectin protein levels, the number of adiponectin secretions, and gene expressions compared between groups were analyzed using a one-way analysis of variance (ANOVA) with Turkey's HSD post hoc test (SPSS v. 23). Values were considered statistically significant differences, which means sharing the different superscript letters when *P* < 0.05, and data were representative of three independent experiments (*n* = 3). Most of the experiments were performed in triplicate.

## 3. Results

### 3.1. LC-MS/MS Chromatograms and Quantification of *O. indicum* Extract

**Figure 1:**
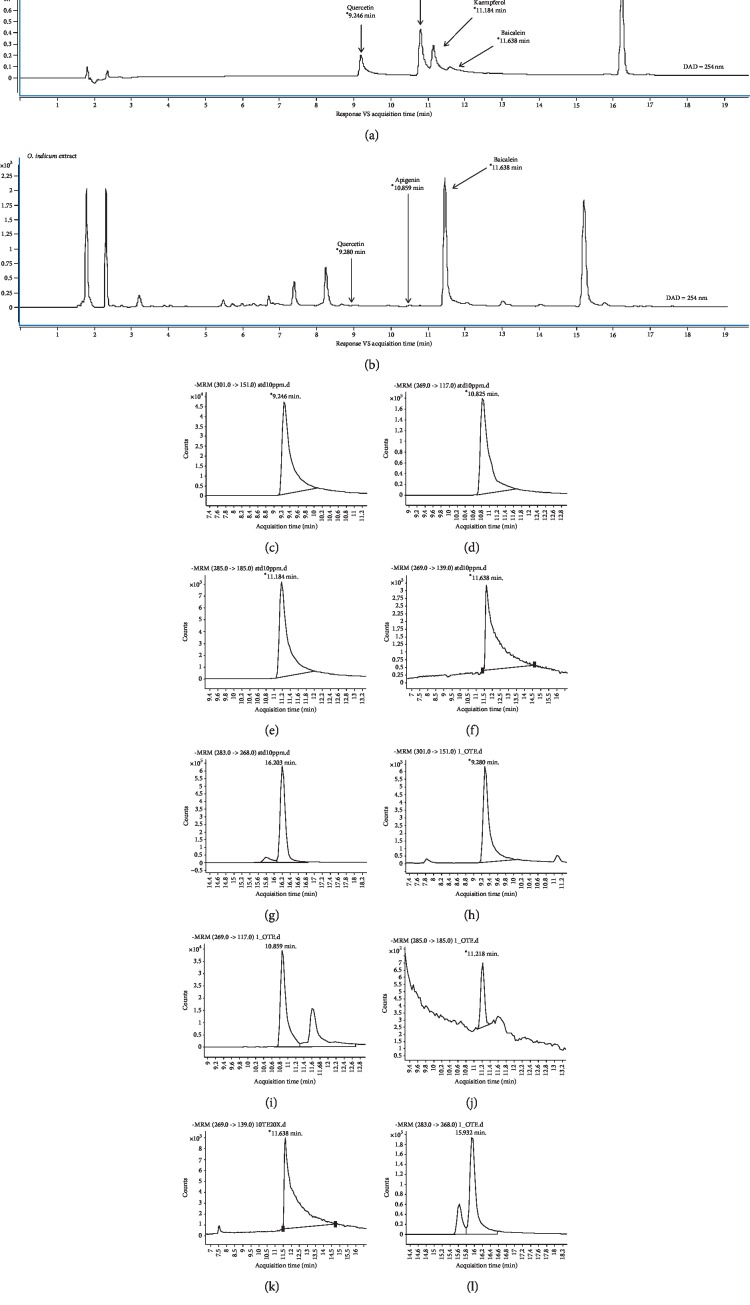
Multiple reaction monitoring (MRM) chromatograms of *Oroxylum indicum* extract. (a) Standard reference compounds. (b) Mixtures of standard reference compounds. (c) Mass spectra of quercetin. (d) Mass spectra of apigenin. (e) Mass spectra of kaempferol. (f) Mass spectra of baicalein. (g) Mass spectra of biochanin A. (h–l) Individual mass spectra of *Oroxylum indicum* extract at different retention time periods corresponding to the mass spectra of quercetin, apigenin, kaempferol, baicalein, and biochanin A, respectively.

The identification of phytochemical compounds in the OIE was performed by using LC-MS/MS.  Figure 1 presents the MRM chromatograms of the OIE compared to the reference compounds, including quercetin (RT = 9.2 min), apigenin (RT = 10.8 min), kaempferol (RT = 11.2 min), baicalein (RT = 11.6 min), and biochanin A (RT = 16.2 min). It was shown that quercetin, apigenin, and baicalein were identified in the OIE (Figure 1(b)). Besides, the OIE also exhibited other prominent peaks at RT of 1.8, 2.2, and 15.2 min. The result from MRM data quantification of 20 mg mL^−1^ of the OIE exhibited 657.01 *μ*g mL^−1^ of baicalein while the amounts of quercetin and apigenin were found to be very low at 1.10 *μ*g mL^−1^ and 1.21 *μ*g mL^−1^, respectively (Table 1). However, kaempferol and biochanin A were not detected in the OIE.

### 3.2. Effect of *O. indicum* Extract during the Transformation of 3T3-L1 Preadipocytes to Adipocytes

**Figure 2:**
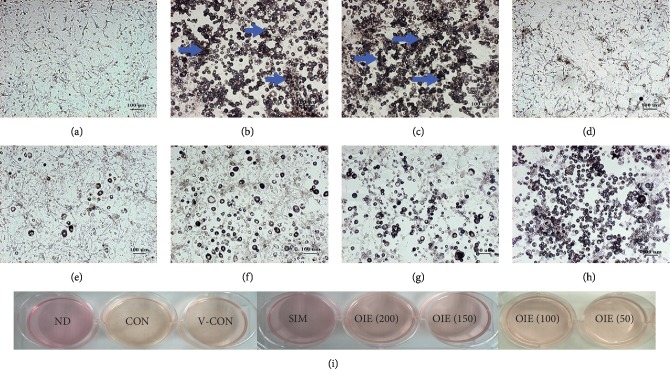
Morphologies of 3T3-L1 preadipocytes after inducing adipogenic inducer and changing the medium color. Arrows indicate lipid droplets. (a) Nondifferentiated cells (ND) or preadipocytes. (b) Untreated adipocytes (CON). (c) Differentiated cells with 0.1% DMSO (V-CON). (d) Differentiated cells with 1.67 *μ*g mL^−1^ simvastatin (SIM). Differentiated cells with OIE at (e) 200 *μ*g mL^−1^; (f) 150 *μ*g mL^−1^; (g) 100 *μ*g mL^−1^; (h) 50 *μ*g mL^−1^. (a–h) Scale bar represents 100 *μ*m, magnification at ×40. (i) The color of the media at day 12.

**Figure 3:**
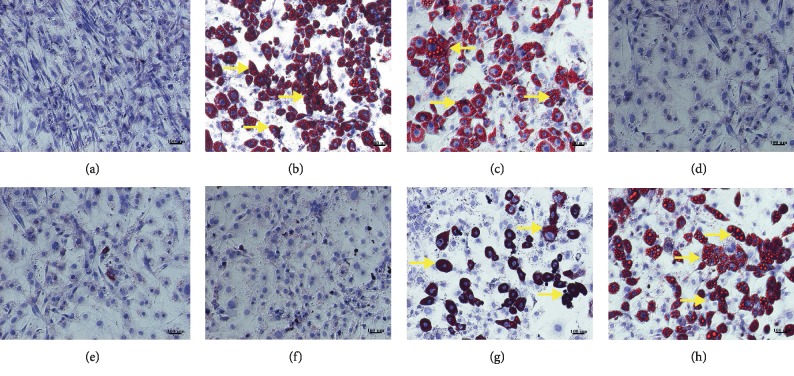
Morphology of 3T3-*μ*L1 preadipocytes and adipocytes after staining with Oil Red O. Lipid droplets were stained in red color (yellow arrows). Nuclei were counterstained with hematoxylin, as indicated in blue color. (a) Nondifferentiated cells (ND) or preadipocytes. (b) Untreated adipocytes (CON). (c) Differentiated cells with 0.1% DMSO (V-CON). (d) Differentiated cells with 1.67 *μ*g mL^−1^ simvastatin (SIM). Differentiated cells with OIE at (e) 200 *μ*g mL^−1^; (f) 150 *μ*g mL^−1^; (g) 100 *μ*g mL^−1^; (h) 50 *μ*g mL^−1^. (a–h) Scale bar represents 100 *μ*m, at 10X magnification.

3T3-L1 preadipocytes displayed fibroblastic morphology, as shown in Figure 2(a) and 3(a). However, on day 10 after 3T3-L1 preadipocytes were differentiated, the cells in the control group (Figures 2(b) and 3(b)) and vehicle control group (Figures 2(c) and 3(c)) were developed to adipocytes resulting in more numerous and larger sizes of intracellular lipid droplets stained in red color (Figure 3). In contrast, it was shown that the cells treated with simvastatin (Figures 2(d) and 3(d)) or the OIE at 200, 150, 100, and 50 *μ*g mL^−1^ (Figures 2(e)–2(h) and 3(e)–3(h), respectively) showed the decrease in number and size of lipid droplets in a dose-dependent manner. Furthermore, at the end of the differentiation process, the media of the control and vehicle control groups were changed to yellow color when compared to those of the nondifferentiated group (Figure 2(i)). However, differentiated cells treated with simvastatin and the OIE at 150 and 200 *μ*g mL^−1^ remained in the same color as that of the nondifferentiated cells. The Oil Red O and hematoxylin staining results (Figure 3) are consistent with the result of medium color changing (Figure 2(i)) suggesting that OIE at high concentrations could reduce the lipid droplet accumulation in adipocytes.

### 3.3. Effect of *O. indicum* Extract on Adiponectin Protein Expression in 3T3-L1 Cells

**Figure 4:**
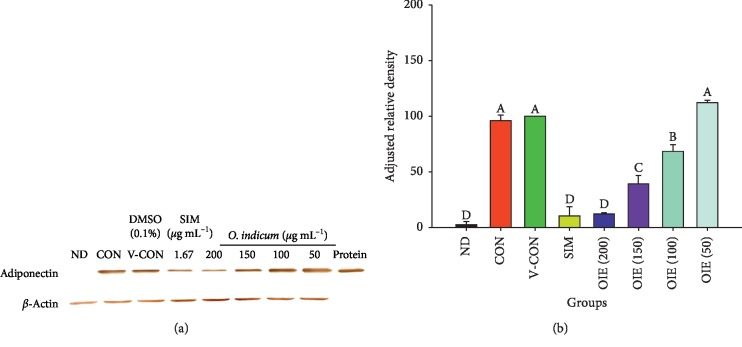
Effect of *Oroxylum indicum* extract (OIE) on adiponectin protein expression. (a) The images of adiponectin protein expression. ND = nondifferentiated cells (preadipocytes); CON = untreated differentiated cells; V-CON = differentiated cells with 0.1% DMSO (vehicle control); OIE (200) = OIE at 200 *μ*g mL^−1^; OIE (150) = OIE at 150 *μ*g mL^−1^; OIE (100) = OIE at 100 *μ*g mL^−1^; OIE (50) = OIE at 50 *μ*g mL^−1^; SIM = simvastatin at 1.67 *μ*g mL^−1^. (b) Comparison of the intensity of adiponectin protein expression among 8 groups. All values are presented as the mean ± SD for three replicates. Means with the different superscript letters are significantly different from each other (ANOVA with Tukey's HSD post hoc test, *P* < 0.05).

Adiponectin is known as an insulin-sensitizing hormone which is produced and secreted from white adipocytes. On day 12, the cells were harvested, and the lysates were subjected to western immunoblotting for adiponectin expression. Adiponectin expressed in 3T3-L1 preadipocytes and adipocytes was identified at 30 kDa. A recombinant human adiponectin protein, which served as the positive control, showed the same molecular weight. The intensities of adiponectin protein bands of OIE at 50 *μ*g mL^−1^ were not significantly different from those of the control and vehicle control groups (*P* > 0.05), although the intensities of OIE at 100, 150, and 200 *μ*g mL^−1^ and simvastatin-treated groups were significantly lower than those of the control and vehicle control groups (*P* < 0.05) (Figures 4(a) and 4(b)).

### 3.4. Effect of *O. indicum* Extract on the Secretion of Adiponectin

**Figure 5:**
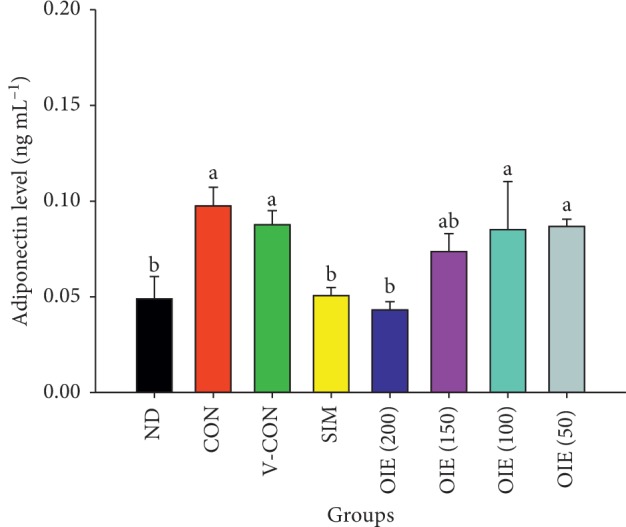
Effect of *Oroxylum indicum* extract (OIE) on the secretion of adiponectin during differentiation. ND = nondifferentiated cells (preadipocytes); CON = untreated adipocytes; V-CON = differentiated cells with 0.1% DMSO (vehicle control); OIE (200) = OIE at 200 *μ*g mL^−1^; OIE (150) = OIE at 150 *μ*g mL^−1^; OIE (100) = OIE at 100 *μ*g mL^−1^; OIE (50) = OIE at 50 *μ*g mL^−1^; SIM = simvastatin at 1.67 *μ*g mL^−1^. All values are presented as the mean ± SD for three replicates. Means with the different superscript letters are significantly different from each other (ANOVA with Tukey's HSD post hoc test, *P* < 0.05).

On day 12, the media were collected for the measurement of levels of adiponectin. The levels of adiponectin secretion were significantly elevated in control, vehicle control, and 50, 100, and 150 *μ*g mL^−1^ of the OIE-treated group compared to the nondifferentiated group (*P* < 0.05; Figure 5). However, the level of adiponectin secretion from adipocytes treated with 200 *μ*g mL^−1^ OIE was significantly lower than that of the control group for approximately 2 times (*P* < 0.05). Adipocytes treated with simvastatin showed a similar decreasing level of adiponectin secretion compared to the 200 *μ*g mL^−1^ of OIE-treated group.

### 3.5. Effect of *O. indicum* Extract on Adiponectin Staining in 3T3-L1 Cells

**Figure 6:**
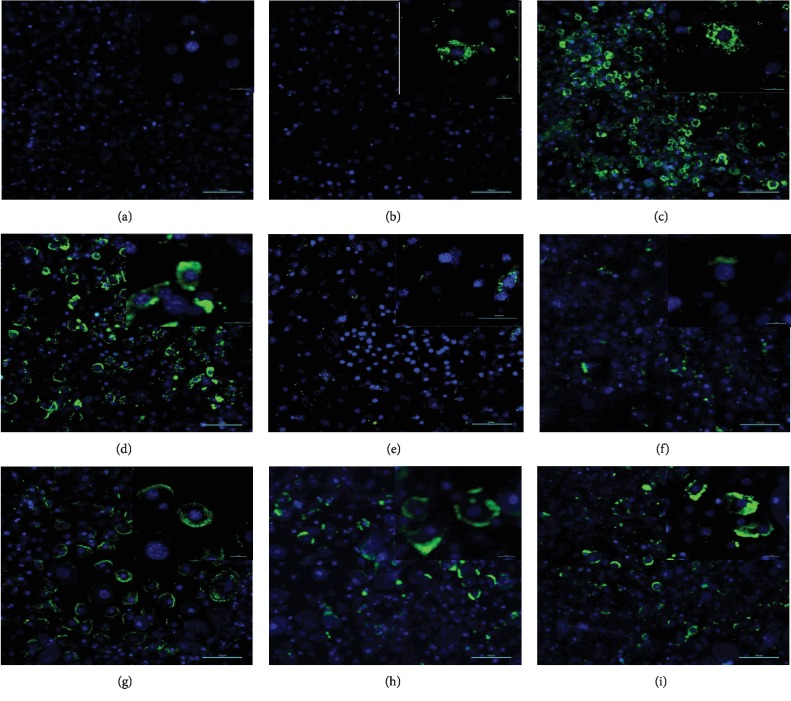
Effect of *Oroxylum indicum* extract (OIE) on the localization of adiponectin in the 3T3-L1 cells. The adiponectin is stained in green color, and nuclei are stained in blue color. (a) Negative control, differentiated cells without adiponectin, shows no staining of the adiponectin. (b) A few staining of the adiponectin in the nondifferentiated cells with adiponectin (ND, preadipocytes). (c) Staining of the adiponectin is observed in the untreated differentiated cells (CON). (d) Differentiated cells treated with 0.1% DMSO (V-CON, vehicle control). (e) Differentiated cells treated with simvastatin at 1.67 *μ*g mL^−1^ (SIM, positive control). Differentiated cells treated with OIE at (f) 200 *μ*g mL^−1^; (g) 150 *μ*g mL^−1^; (h) 100 *μ*g mL^−1^; (i) 50 *μ*g mL^−1^. Scale bar: 100 *μ*m.

Adiponectin was found to be localized in the cytoplasm of the 3T3-L1 cells (Figure 6).  Figure 6(a) shows fluorescent staining of nuclei in differentiated cells without adiponectin staining (negative control). A few localization of the adiponectin could be observed in the nondifferentiated cells with adiponectin (ND) (Figure 6(b)). The adiponectin was detected markedly in the control (CON) (Figure 6(c)) and vehicle control groups (V-CON) (Figure 6(d)). In contrast, when the cells were treated with simvastatin (SIM, a positive control) and 50 to 200 *μ*g mL^−1^ OIE during differentiation (Figures 6(e)–6(i)), simvastatin and the OIE at 200 *μ*g mL^−1^ caused the lowest localization of adiponectin in the cells (Figures 6(e) and 6(f)).

### 3.6. Effect of *O. indicum* Extract on mRNA Expression in 3T3-L1 Cells

**Figure 7:**
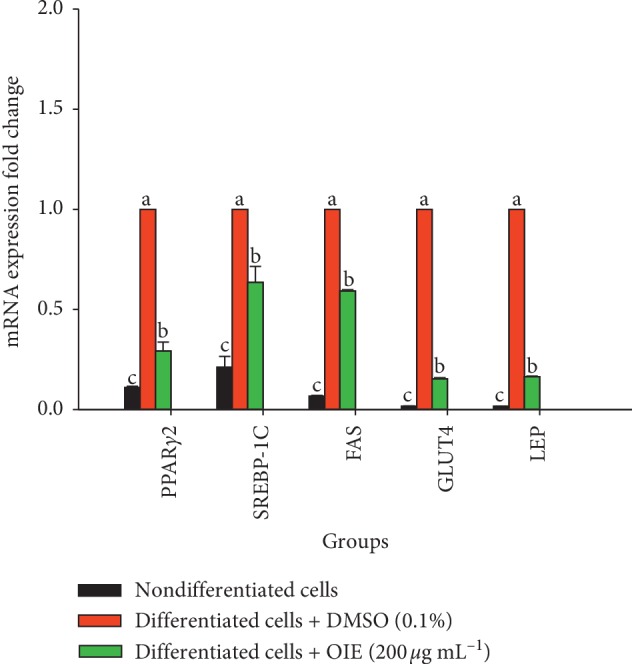
Effect of *Oroxylum indicum* extract (OIE) on mRNA expression of PPAR*γ*2, SREBP-1C, FAS, GLUT4, and LEP in 3T3-L1 cells. The expression values were normalized to the *β*-actin. The results are presented as the mean ± SD for three triplicate. Means with the different superscript letters are significantly different from each other (ANOVA with Tukey's HSD post hoc test, *P* < 0.05).

To clarify the mechanism underlying the antiadipogenesis of the OIE, expression levels of mRNA of PPAR*γ*2, SREBP-1C, FAS, GLUT4, and LEP were studied. The vehicle control group exhibited significantly increased PPAR*γ*2 and SREBP-1C mRNA expression by 10 folds and 5 folds, respectively (*P* < 0.05) compared to the nondifferentiated group. A similar pattern was observed in FAS, GLUT4, and LEP by more than 10 times in the vehicle control group compared to the nondifferentiated group (Figure 7). The mRNA expression level in the cells treated with 200 *μ*g mL^−1^ OIE was significantly decreased approximately 1/2 times compared to the vehicle control group (*P* < 0.05). These results suggest that OIE can inhibit adipogenesis via inhibition of the change of the mRNA level.

## 4. Discussion

Adipogenesis is a complex process of adipocyte differentiation and development, leading to an increased amount of adipose tissue mass [22]. Recently, it was discovered that inhibition of adipogenesis had been a potential target for plant extracts and their bioactive compounds for the prevention and treatment of obesity [23]. 3T3-L1 cells are a major preadipocyte cell line model that has been used for adipogenesis in many studies [24, 25]. The specific character of these cells is when it is treated with the appropriate hormone, it can differentiate to adipocyte lineage and also change the expression of numerous genes involved in adipogenesis [26].

The OIE is a crude compound extracted from the fruit of *O. indicum* plants. Evaluation of the phytochemical composition of OIE using the LC-MS/MS technique revealed that the OIE contained quercetin, apigenin, and baicalein. The MRM mode, which was used for the data quantification, showed that 657.01 *μ*g mL^−1^ of baicalein was the most dominant flavone found in 20 mg mL^−1^ of OIE while very low amounts of quercetin and apigenin were identified in the OIE (1.10 *μ*g mL^−1^ and 1.21 *μ*g mL^−1^, respectively). However, kaempferol and biochanin A were not detected in the OIE. These results are in accordance with the previous report that baicalein is the major component of methanolic extract of the fruits of *O. indicum* [14]. Our previous report indicated that the OIE showed an antiadipogenesis effect in 3T3-L1 adipocytes and had an impact on lipid and carbohydrate biomolecular changes of the cell [13]. In the present study, we found that the OIE inhibited the differentiation of preadipocytes and retained the fibroblastic morphology almost the same as those of the preadipocyte stage while the nontreated adipocytes and vehicle control showed numerous lipid droplets in their cytoplasm. Also, the media of nontreated adipocytes or vehicle control were changed to yellow color. However, no significant change in the color of the media was observed in preadipocytes and adipocytes treated with simvastatin or the OIE. The corresponding results of the Oil Red O and hematoxylin staining and medium color changing provide evidence that OIE at high concentrations could lower the lipid droplet accumulation in differentiated adipocytes. The possible explanation is that adipocytes may have an active metabolic stage and secrete numerous factors of adipokine.

At the molecular level, elevations in protein localization, secretion, and mRNA expression levels of adiponectin were observed in control and vehicle control adipocytes, which were related to adipogenesis and cellular lipid accumulation. These findings are consistent with those of Ikeda et al. that the mRNA expression of adiponectin in 3T3-L1 cells increases throughout the maturation process of adipocytes [27]. Apart from this, Fu et al. reported that adiponectin caused the proliferation and differentiation of preadipocytes to adipocytes through promoting prolonged transcriptional factors in the process of cell differentiation [18]. Our findings led us to believe that these genes can be used as adipocyte maturation markers and can help to maintain the size of the adipocytes. However, adipocytes treated with 200 *μ*g mL^−1^ of OIE were significantly decreased in localization and secretion of the adiponectin protein and mRNA expression compared to the control adipocytes. The possible explanation can be that the OIE delays the progression of cell proliferation and causes adiponectin levels to be lower than those of the control group. In particular, it was shown that 12.5 *μ*M of baicalein, a flavone presented in the fruit of OIE, was able to block the G0/G1 phase of the 3T3-L1 cell cycle induction to adipocytes at 24 h [28]. Moreover, numerous studies revealed that the inhibitory effect of the crude extract or their compound on the differentiation of adipocytes is caused by the inhibition of the cell cycle [29, 30].

*In vitro* studies revealed that adipogenesis occurred in 2 major steps, including the recruitment and proliferation of preadipocytes followed by their subsequent differentiation into mature fat cells [31]. Differentiation is companied by the sequential expression of key transcription factors that direct the adipogenic program. Peroxisome proliferator-activated receptor-gamma 2 (PPAR*γ*2) is playing a crucial role in the differentiation program. The mechanism of PPAR*γ* was initiated by ligands, which could be a hormone, a free fatty acid, fatty acid, derivative, or synthetic drugs, which bound to the nuclear receptor. The PPAR*γ* then formed heterodimers with the retinoid X receptor (RXR), thereby increasing adipogenic genes and protein expression, including leptin and GLUT4 [32]. SREBP-1c is a transcriptional factor mediating the expression levels of genes involved in fatty acid metabolism and de novo lipogenesis [33]. An *in vitro* study found that overexpression of SREBP-1c in cultured preadipocytes activated genes involved in fatty acid and triglyceride syntheses such as acetyl CoA carboxylase (ACC) and fatty acid synthase (FAS) [34]. In the present study, we selected the most effective concentration of the OIE for studying the mRNA expression of the adipocyte gene. Our results showed that 200 *μ*g mL^−1^ of OIE significantly prevented upregulation of PPAR*γ*2 and SREBP-1c genes compared to the control mature adipocytes. It is noteworthy that PPAR*γ* was essential for normal adipocyte differentiation and PPAR*γ* knockdown largely prevented adipogenic differentiation of 3T3-L1 cells [35]. Thus, OIE could mediate lipogenesis either via the delay of cell cycle progression or modulation of PPAR*γ* and SREBP-1c genes. Our study also detected the expression of GLUT4, which facilitates glucose transportation into the cells, and FAS, a product of de novo synthesis, which is the crucial source for the intracellular triglyceride synthesis. The results indicated that adipocytes treated with the OIE significantly inhibited an increase in both GLUT4 and FAS expression along with PPAR*γ*, SREBP-1c, and leptin genes compared to the untreated adipocytes.

## 5. Conclusions

In summary, these findings provide evidence that the potential antiadipogenesis mechanism of the OIE can be the downregulation of PPAR*γ*2 and lipogenic genes controlling adipogenesis, including SREBP-1c, FAS, and GLUT4, leading to diminishing adipokines marker secretion from adipocytes (adiponectin and leptin). Thus, the OIE may be developed as a new natural resource for a lipid-lowering drug.

## Figures and Tables

**Table 1:** Quantification of selected compounds in *O. indicum* extract (OIE).

Compounds	Quercetin (301-->151)	Apigenin (269-->117)	Kaempferol (285-->185)	Baicalein (269-->139)	Biochanin A (283-->268)
OIE (20 mg mL^−1^)<	1.10 *μ*g mL^−1^	1.21 *μ*g mL^−1^	ND (detection limit = 0.55 *μ*g mL^−1^)	657.01 *μ*g mL^−1^	ND (detection limit = 0.11 *μ*g mL^−1^)

ND = not detected.

## Data Availability

The data sets used and analyzed during this study are available from the corresponding author upon reasonable request.
